# Phospholysine phosphohistidine inorganic pyrophosphate phosphatase suppresses insulin‐like growth factor 1 receptor expression to inhibit cell adhesion and proliferation in gastric cancer

**DOI:** 10.1002/mco2.472

**Published:** 2024-01-30

**Authors:** Zihao Zhang, Xu Wang, Yuan Liu, Hao Wu, Xingyu Zhu, Chunshui Ye, Huicheng Ren, Wei Chong, Liang Shang, Leping Li

**Affiliations:** ^1^ Department of Gastrointestinal Surgery Shandong Provincial Hospital Shandong University Jinan Shandong China; ^2^ Department of General Surgery Zhongshan Hospital Fudan University Shanghai China; ^3^ Department of Anesthesiology Shandong Provincial Hospital Affiliated to Shandong First Medical University Jinan Shandong China; ^4^ Department of Gastrointestinal Surgery Shandong Provincial Hospital Affiliated to Shandong First Medical University Jinan Shandong China; ^5^ Department of General Surgery Peking Union Medical College Peking Union Medical College Hospital Chinese Academy of Medical Sciences Beijing China; ^6^ Medical Science and Technology Innovation Center Shandong First Medical University & Shandong Academy of Medical Sciences Shandong China; ^7^ Key Laboratory of Engineering of Shandong Province Shandong Provincial Hospital Jinan Shandong China

**Keywords:** cell adhesion, gastric cancer, IGF1R, LHPP, proliferation

## Abstract

Phospholysine phosphohistidine inorganic pyrophosphate phosphatase (LHPP) has recently emerged as a novel tumor suppressor. Researchers have observed that LHPP plays a crucial role in inhibiting proliferation, growth, migration, invasion, and cell metabolism across various cancers. Nevertheless, the specific functions and underlying mechanisms of LHPP as a tumor suppressor in gastric cancer (GC) require further exploration. The expression of LHPP was assessed in human GC specimens and cell lines. Various assays were employed to evaluate the impact of LHPP on GC cells. RNA sequencing and Gene Set Enrichment Analysis were conducted to unravel the mechanism through which LHPP regulates GC cell behavior. Additionally, xenograft nude mouse models were utilized to investigate the in vivo effects of LHPP. The findings indicate that LHPP, functioning as a tumor suppressor, is downregulated in both GC tissues and cells. LHPP emerges as an independent risk factor for GC patients, and its expression level exhibits a positive correlation with patient prognosis. LHPP exerts inhibitory effects on the adhesion and proliferation of GC cells by suppressing the expression of insulin‐like growth factor 1 receptor (IGF1R) and modulating downstream signaling pathways. Consequently, LHPP holds potential as a biomarker for targeted therapy involving IGF1R inhibition in GC patients.

## Graphical abstract

LHPP, downregulated in gastric cancer, can be used as an independent risk factor for gastric cancer patients, and its expression level is positively correlated with the prognosis of patients. LHPP inhibits gastric cancer cells' proliferation, migration, invasion, and adhesion. The dysregulation of LHPP expression leads to the upregulation of IGF1R, thereby activating the downstream ITGB1–FAK–SRC/YAP–c‐MYC signaling pathway, resulting in the development and progression of gastric cancer.

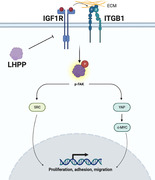

## 1 INTRODUCTION

Gastric cancer (GC) is a prevalent malignant tumor originating from the epithelial cells of the gastric mucosa, ranking fifth globally in incidence and third in associated cancer‐related deaths.^1^ The incidence of GC varies significantly worldwide, with decreasing rates observed in Western Europe and North America, but persistently high rates in East Asia. Early‐stage GC often lacks obvious symptoms, whereas advanced‐stage GC manifests with symptoms like abdominal discomfort and weight loss. Consequently, many patients are diagnosed only at advanced stages, with a higher likelihood of recurrence and metastasis to organs such as the liver and peritoneum.^2^ Currently, surgery and systemic chemotherapy are the primary treatment modalities for GC patients. Despite the increasing use of immunotherapy and targeted therapy, the 5‐year survival rate for GC patients remains low.^3^ Therefore, exploring more effective treatment methods and biomedical targets for GC holds significant clinical implications for improving prognosis and extending overall survival.

Phosphatases, particularly serine, threonine, and tyrosine phosphatases, regulate cellular behavior and signal transduction by dephosphorylating substrates. Research indicates that phosphatases play a crucial role in tumor development. Due to the instability of histidine phosphorylation states and limitations in detection techniques, research on histidine phosphatases and phosphorylation remains limited. As research progresses, attention has turned to phospholysine phosphohistidine inorganic pyrophosphate phosphatase (LHPP). Encoded by the LHPP gene on chromosome 10, LHPP removes phosphate groups from protein histidines. It is expressed in various tissues such as the brain, thyroid, liver, and kidneys.^4^ Initial research revealed a close connection between LHPP and neurological disorders and abnormalities.^5^ Subsequent studies found that LHPP expression is reduced in liver cancer tissues, and overexpression of LHPP inhibits the progression and severity of liver cancer in animal models, suggesting its tumor‐suppressive role. Furthermore, LHPP expression levels are positively correlated with patients' overall survival and disease‐free survival, indicating its potential as a prognostic factor for liver tumors.^6^ In recent years, an increasing number of studies have found that LHPP dysregulation is associated with the development of various tumors, such as oral squamous cell carcinoma, thyroid cancer, hepatocellular carcinoma, and non‐small cell lung cancer.^7^, ^8^, ^9^, ^10^ LHPP not only inhibits tumor cell proliferation, metastasis, and invasion but also promotes apoptosis and regulates tumor cell metabolism. At present, the specific role of LHPP in the occurrence and development of GC remains to be further explored.

Cell adhesion plays a crucial role in multicellular organisms, connecting cells to the extracellular matrix and linking cells together through the cytoskeleton to facilitate signal transduction.^11^ Cell adhesion also plays a vital role in tumor metastasis and recurrence. Many studies have shown that typical cell adhesion markers such as E‐cadherin, N‐cadherin, β‐catenin, and cell‐extracellular matrix adhesion markers such as integrins, focal adhesion kinase (FAK), and Non‐Receptor Tyrosine Kinase (SRC) protein, have a significant impact on the characteristics and behavior of various tumor cells.^12^, ^13^ Integrins are the primary mediators of cell–matrix adhesion. In normal colonic mucosal epithelium, α6Aβ4 integrin is predominant, while in colon cancer cells, the expression of α6Aβ4 integrin significantly increases, promoting colon cancer cell proliferation.^14^ FAK, a kind of protein tyrosine kinase is involved in multiple signaling pathways related to tumor proliferation and metastasis, and it activates the TGF‐β signaling pathway to promote breast cancer bone metastasis.^15^ SRC is overexpressed and hyperactivated in various solid tumors and regulates cell functions such as proliferation, migration, invasion, and adhesion by interacting with estrogen receptors, vascular endothelial growth factor receptors, and other factors. It is considered an important target for cancer therapy.^16^ Understanding GC metastasis and malignant progression necessitates a focus on studying cell adhesion capacity.

In the present study, we found the LHPP expression was lower in GC than in adjacent normal tissues as indicated by online databases such as The Cancer Genome Atlas Program (TCGA) and Gene Expression Omnibus (GEO) databases, along with samples from 90 GC patients. Next, we evaluated the role of LHPP in the proliferation and metastasis of GC cells through various assays. Gene Set Enrichment Analysis (GSEA) analysis and RNA sequencing revealed differential expression of cell adhesion‐related gene sets between LHPP overexpression GC cells and control cells. The cell adhesion ability of GC cells was detected by attachment and detachment assays. Additionally, we identified insulin‐like growth factor 1 receptor (IGF1R) as a downstream target of LHPP and demonstrated that the antitumor effects of LHPP on GC cells were partially reversed upon IGF1R knockdown. Our findings underscore the significant role of LHPP in regulating IGF1R for its antitumor effects in GC.

## 2 RESULTS

### 2.1 LHPP is downregulated in GC tissues and is a potential prognosis biomarker for GC patients

**FIGURE 1:**
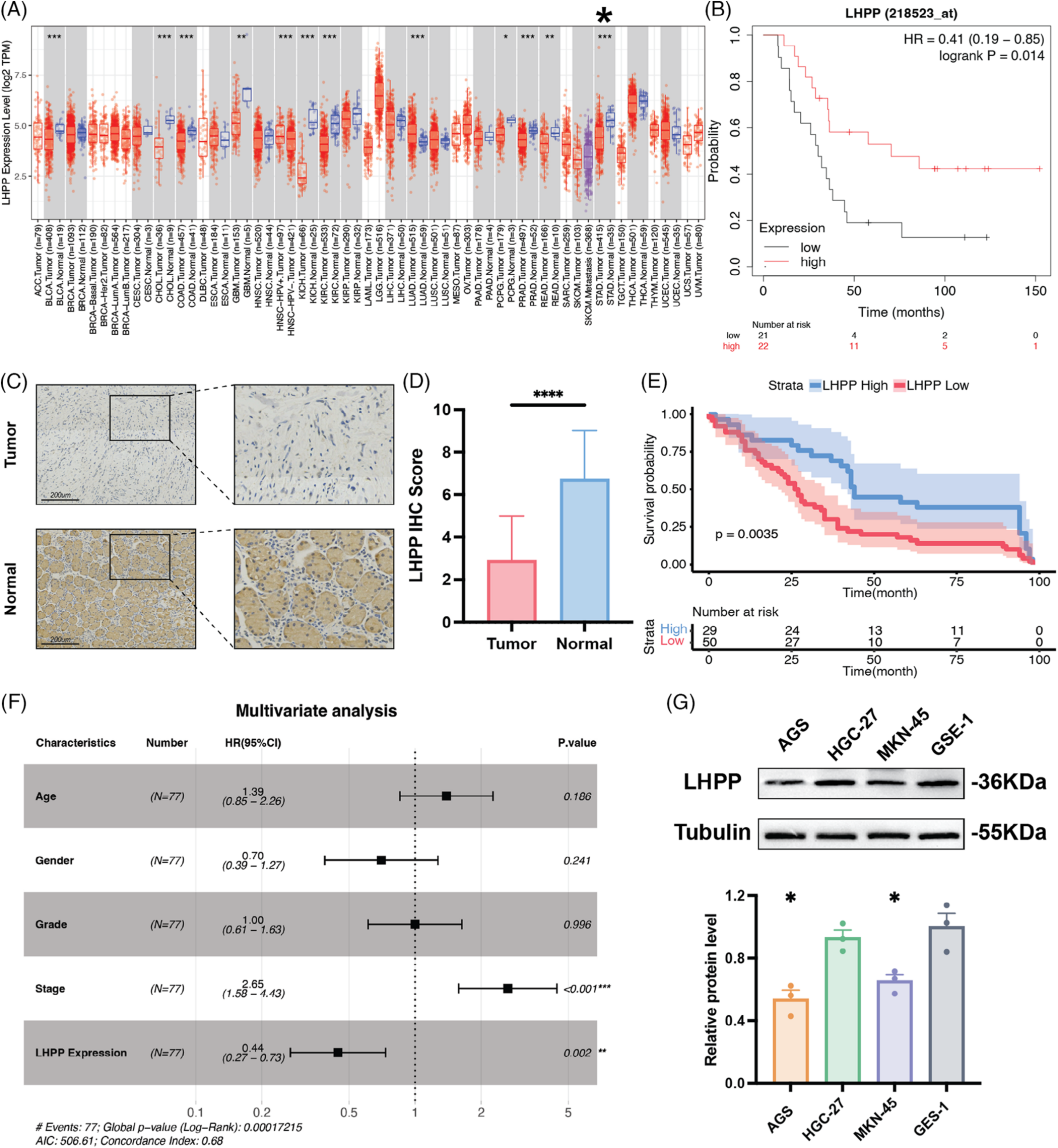
LHPP is downregulated in GC tissues and is a potential prognosis biomarker for GC patients. The differential expression of LHPP between GC and normal tissue according to the (A) TCGA database and (C and D) 89 GC patients are shown. (B and E) Prognosis of GC patients between high LHPP expression groups and low LHPP expression groups. (F) Multivariate analysis of prognostic factors in GC patients. (G) LHPP expression in GC cell lines, AGS, HGC‐27, MKN‐45, and normal mucosal cells, GES‐1. The data are presented as mean ± SD. ^*^ *p* < 0.05, ^**^ *p* < 0.01, ^***^ *p* < 0.001, ^****^ *p* < 0.0001.

The expression of LHPP in GC was investigated using data from the TCGA database. The analysis revealed a significant downregulation of LHPP expression in GC tissues compared with normal tissues (Figure 1A). Immunohistochemistry (IHC) was used to measure the LHPP expression in 90 GC samples along with their paired normal adjacent tissues. The IHC scores indicated a substantial decrease in LHPP expression in GC samples (Figures 1C and D). Kaplan–Meier analysis was used to explore the relationship between LHPP and the prognosis of GC patients. A lower LHPP expression was significantly associated with shorter overall survival according to the GEO (GSE22377) database (Figure 1B). Consistently, GC patients with lower LHPP expression exhibited markedly reduced overall survival compared with those with higher LHPP expression (Figure 1E). To further validate the prognostic significance of LHPP expression, univariate and multivariate Cox analyses were performed to identify factors influencing the prognosis of GC patients. In univariate COX analysis, the following factors were significantly related to OS: stage (HR: 2.1, 95%CI: 1.3–3.4, *p* = 0.0015) and LHPP expression (HR: 0.52, 95%CI: 0.32–0.83, *p* = 0.0064) (Table S1). According to the multivariate COX analysis, stage (HR: 2.65, 95%CI: 1.58–4.43, *p* > 0.001) and LHPP expression (HR: 0.44, 95%CI: 0.27–0.73, *p* > 0.002) were associated greatly with LHPP expression (Figure 1F). In conclusion, LHPP expression might serve as an independent biomarker for predicting GC prognosis.

### 2.2 Overexpressing LHPP inhibits GC cell growth and proliferation

**FIGURE 2:**
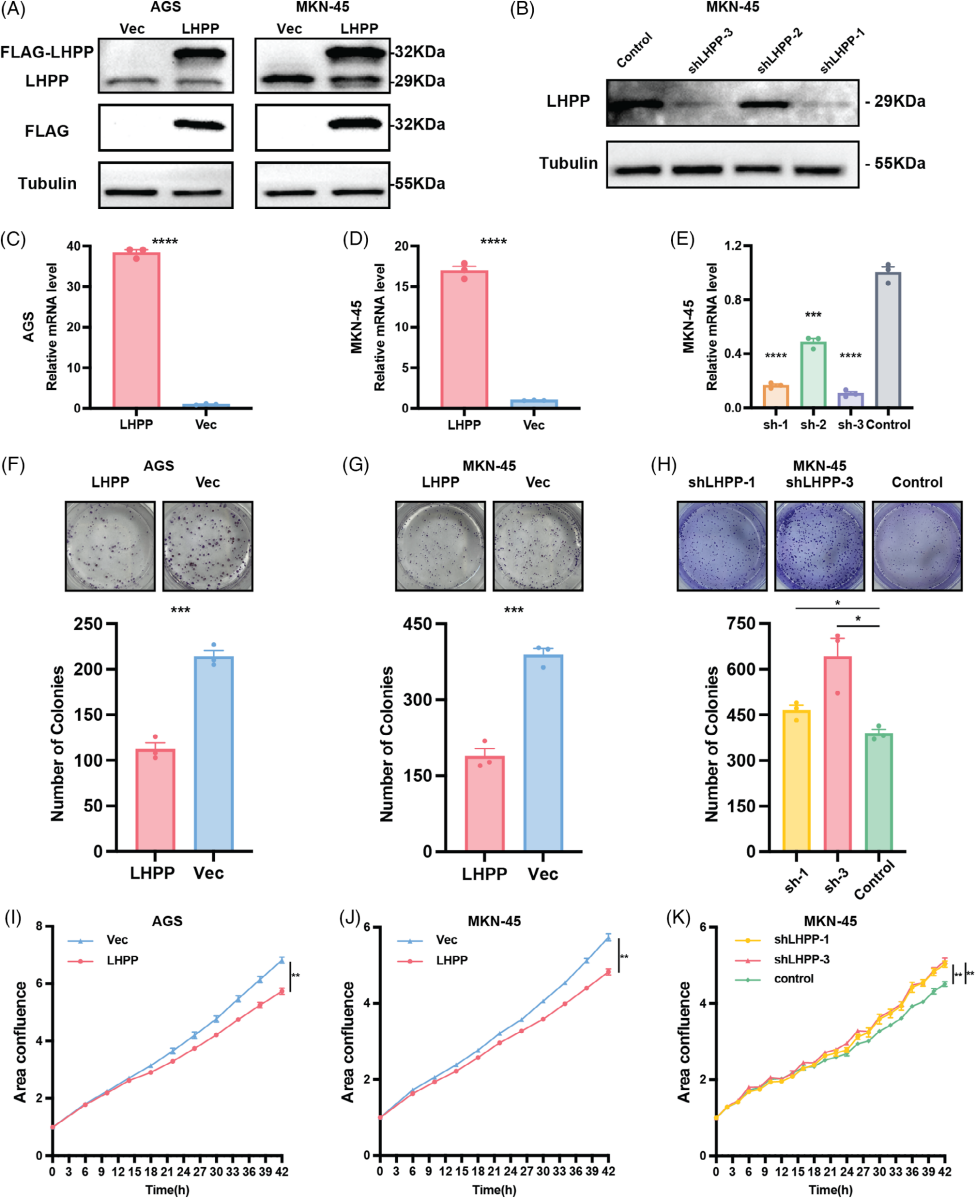
LHPP inhibits the proliferation and growth of GC cells. The transfection efficiency of LHPP overexpression in AGS and MKN‐45 was confirmed by western blot (A) and qRT‐PCR (C and D). The transfection efficiency of LHPP knockdown in MKN‐45 was confirmed by western blotting (B) and qRT‐PCR (E). GAPDH was used as an internal control. The effect of LHPP overexpression (F and G) and knockdown (H) on the colony‐forming capacity of GC cells. The number of clones was counted as shown in the panels below. Phase area confluence was performed to determine the proliferation of LHPP overexpression (I and J) and LHPP knockdown (K) GC cells at the indicated time points after seeding. The data are presented as mean ± SD. ^*^ *p* < 0.05, ^**^ *p* < 0.01, ^***^ *p* < 0.001, ^****^ *p* < 0.0001.

The expression of LHPP in GC cell lines and gastric mucosal epithelial cells was measured by Western blot. The findings demonstrated that GC cells, particularly AGS and MKN‐45 cell lines, were lower than gastric mucosal epithelial cells in terms of LHPP expression (Figure 1G). LHPP overexpression and knocking down GC cell lines were constructed to study the role of LHPP in GC cells. LHPP‐OE LVs and LHPP‐NC LVs were transfected to AGS and MKN‐45 cell lines. Protein and mRNA levels were then measured. Compared with the control group, mRNA and protein levels of LHPP were significantly up‐regulated in LHPP overexpression groups (Figures 2A, C, and D). Meanwhile, LHPP‐sh#1/2/3 LVs and LHPP‐control LVs were transfected into MKN‐45 cell lines. Compared with the control group, mRNA and protein levels of LHPP were significantly downregulated in LHPP‐sh#1 and LHPP‐sh#3 groups (Figures 2B and E). Colony formation assays and live cell analysis were performed to test the proliferation of GC cells. The colony formation assays revealed a decrease in the number of clone formations upon LHPP overexpression following incubation (Figures 2F and G). Furthermore, live cell analysis demonstrated that elevated LHPP expression led to a reduction in phase area confluence within the LHPP‐overexpressing cell lines (Figures 2I and J). Conversely, the knockdown of LHPP resulted in increased clone formations and promoted phase area confluence (Figures 2H and K). These findings collectively indicate that LHPP can impede proliferation in GC cells.

### 2.3 LHPP inhibits GC cell migration and invasion

**FIGURE 3:**
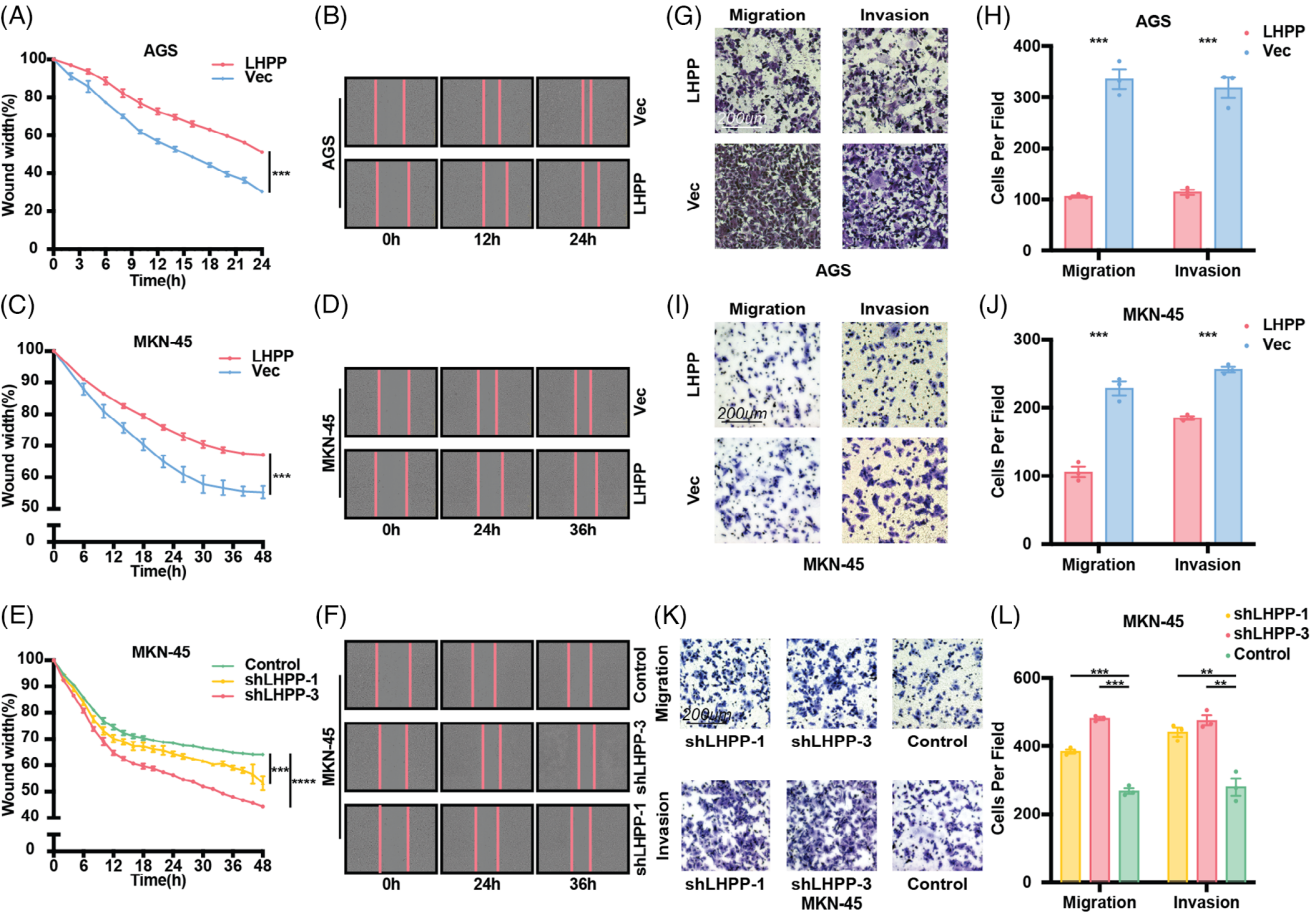
LHPP inhibits the migration and invasion ability of GC cells. Effects of LHPP overexpression (A–D) or LHPP knockdown (E–F) on GC cell migration detected by wound‐healing assays at the indicated time points after scratching. Wound healing was measured by IncuCyte S3 and analyzed by GraphPad Prism 9 software. Migration and invasion ability of LHPP overexpression (G–J) or LHPP knockdown (K and L) GC cells were evaluated by Transwell assay at 24 h. Representative images of migration were captured at 24 h. The data are presented as mean ± SD. ^**^ *p* < 0.01, ^***^ *p* < 0.001, ^****^ *p* < 0.0001.

Wound healing assay and transwell assay were performed to test the migration and invasion of GC cells. It was observed that LHPP overexpression led to a decreased migration rate of GC cells (Figures 3A–D). Conversely, LHPP knockdown notably accelerated the migration of GC cells (Figures 3E and F). Additionally, the count of infiltrating GC cells was diminished in the LHPP overexpression group as compared with the control group, whereas LHPP knockdown resulted in an increased count of infiltrating GC cells (Figures 3G–L). These experiments suggest that LHPP inhibits the migration and invasion of GC cells.

### 2.4 Bioinformatics analysis and experiment assays proved LHPP inhibits GC cell adhesion

**FIGURE 4:**
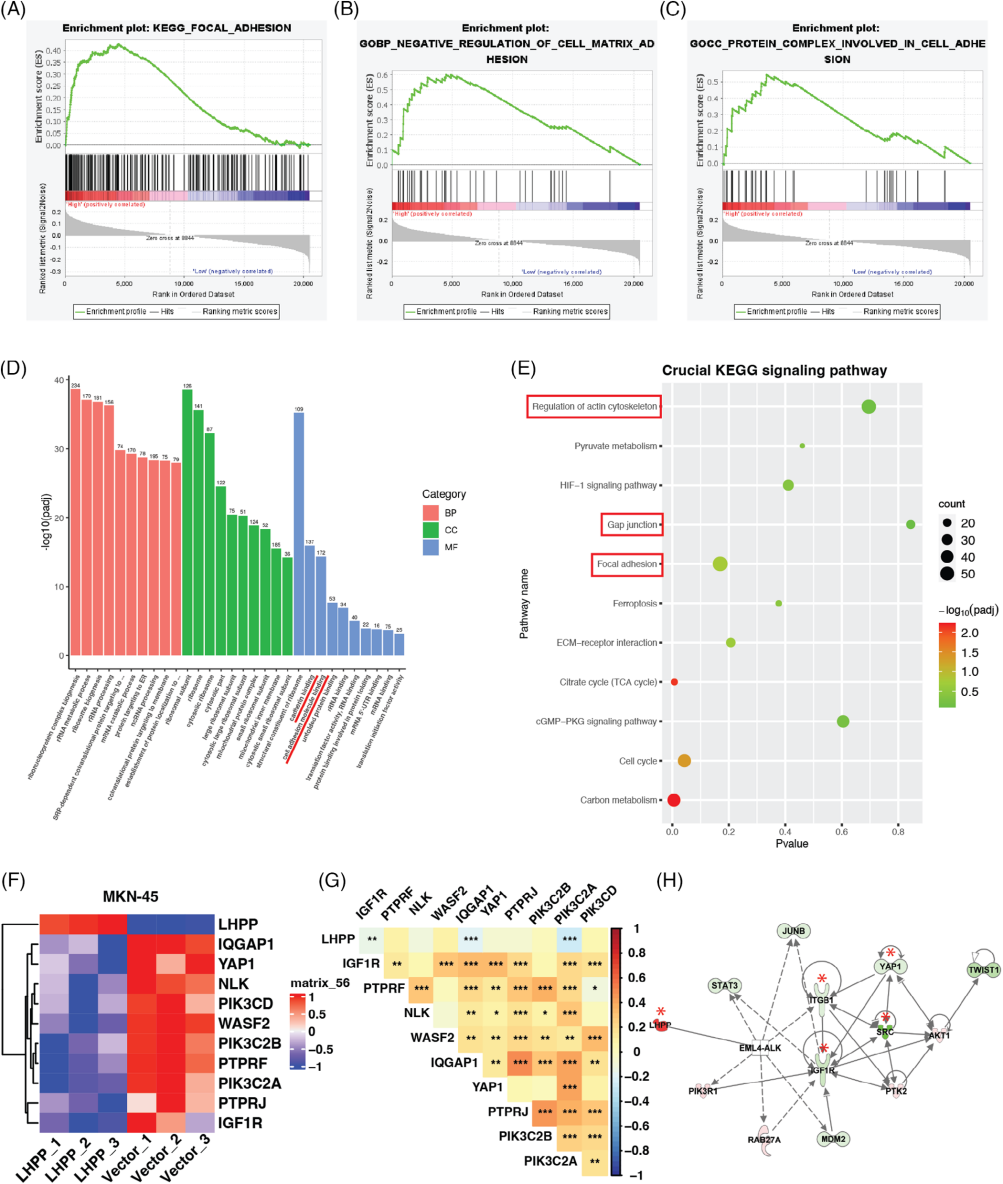
RNA sequencing and bioinformatics analysis predict LHPP inhibits GC cells adhesion. GSEA revealing focal adhesion(A), negative regulation of cell–matrix adhesion(B), and protein complex involved in cell adhesion(C) gene sets were regulated by LHPP expression. RNA sequencing results showed adhesion‐related molecular function (D) and signaling pathway (E) that LHPP overexpression regulated. Downstream DEGs were screened according to RNA sequencing (F) and gene correlation between LHPP and downstream DEGs were analyzed based on the TCGA database (G). IPA analysis revealed adhesion‐related gene LHPP affected (H). ^*^ *p* < 0.05, ^**^ *p* < 0.01, ^***^ *p* < 0.001.

**FIGURE 5:**
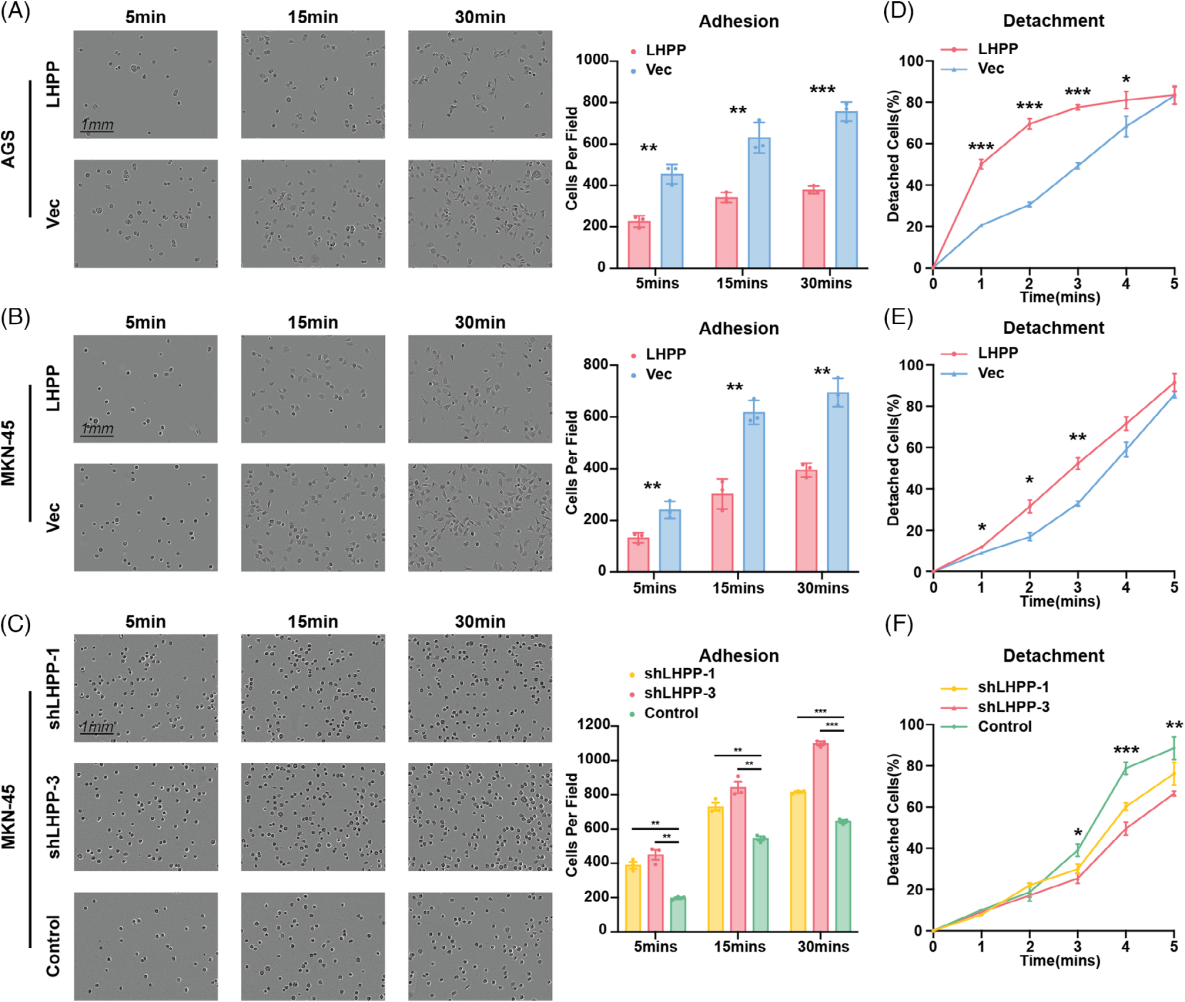
Attachment and detachment assays prove LHPP suppresses the GC adhesion. Cell attachment ability of LHPP overexpression (A and B) or LHPP knockdown (C) GC cells was evaluated by adhesion assays at the indicated time points after seeding. The number of cells was counted as shown in the right panels. Cell detachment ability of LHPP overexpression (D and E) or LHPP knockdown (F) GC cells was assessed by Detachment assays at the indicated time points after trypsinization. The data are presented as mean ± SD. ^*^ *p* < 0.05, ^**^ *p* < 0.01, ^***^ *p* < 0.001.

The samples in ACRG were divided into two groups with high expression of LHPP and low expression of LHPP, and GSEA enrichment analysis was performed on the two groups to analyze the function LHPP could affect. Results showed LHPP could regulate cell adhesion‐related functions, focal adhesion, negative regulation of cell–matrix adhesion, and protein complex involved in cell adhesion (Figures 4A–C). RNA sequencing with LHPP‐overexpressed GC cells and control cells was performed to screen DEGs and functional change. The GO and KEGG analyses proved that cell adhesion functions and pathways were affected, cell adhesion molecule binding, focal adhesion, and so on (Figures 4D and E). The DEGs in the RNA sequencing results intersected with the adhesion‐related pathways in KEGG. Six genes, PTPRF, NLK, WASF2, IQGAP1, IGF1R, and PTPRJ were downregulated after LHPP overexpression (Figure 4F). Besides, key genes in the PI3K‐AKT signaling pathway, proved to be regulated by LHPP, were differentially expressed between LHPP overexpressing and control groups, which confirmed the quality of the RNA sequencing results. According to the TCGA database, the LHPP was inversely related to IGF1R, IQGAP1, and PIK3C2A (Figure 4G). The application of ingenuity pathway analysis (IPA) to the RNA sequencing data revealed a significant downregulation of adhesion‐related genes, notably IGF1R, ITGB1, SRC, and Yes1 Associated Transcriptional Regulator (YAP1), especially in LHPP overexpressing GC cells as compared with the control cells (Figure 4H). Therefore, adhesion ability, detected by attachment and detachment assay, ought to be evaluated, to verify the above findings. The adhesion assay revealed a notable reduction in the number of cells adhering to plates in the LHPP overexpressing groups, as compared with the control group (Figures 5A and B). Conversely, in the LHPP knockdown group, a greater number of cells were found attached to plates than in the control groups (Figure 5C). Turning to the detachment assays, an increased number of cells were observed to detach from plates after trypsinization in the LHPP overexpression group compared with the LHPP control group (Figures 5D and E). Simultaneously, a greater proportion of cells remained on the plates after trypsinization in the LHPP knockdown groups compared with the LHPP control group (Figure 5F). Based on these results, it is easy to conduct that the expression of LHPP affects the adhesion ability of GC cells.

### 2.5 LHPP regulates ITGB1/FAK/YAP signaling pathway via interacting with IGF1R

**FIGURE 6:**
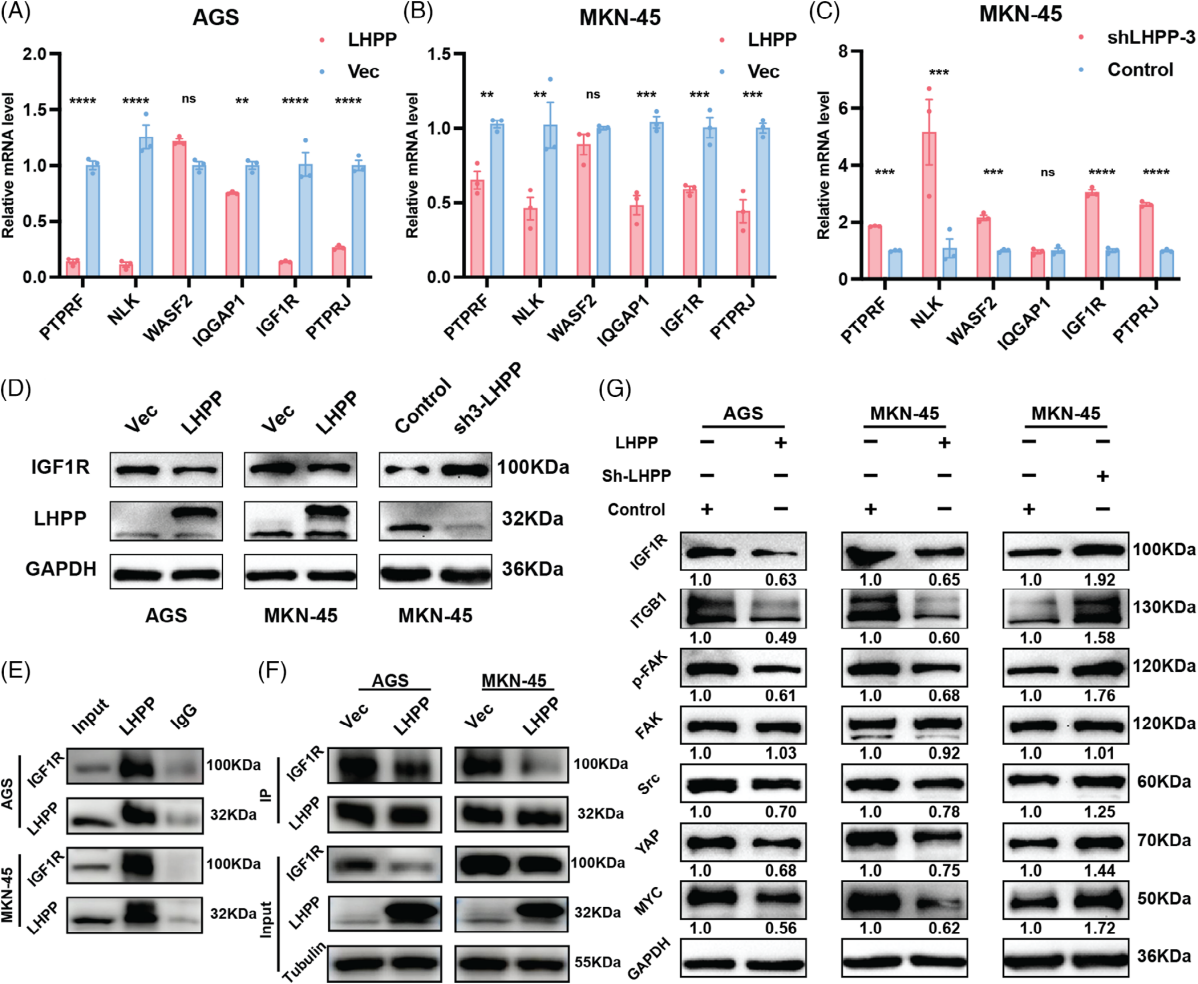
LHPP regulates ITGB1/FAK/YAP signaling pathway way via interacting with IGF1R. Potential downstream genes RNA expression level in LHPP overexpression (A and B) and LHPP knockdown (C) GC cells. The IGF1R protein level in LHPP overexpression and LHPP knockdown GC cells (D). Co‐IP was performed to detect the binding between LHPP and IGF1R (E). The LHPP and IGF1R interaction difference between LHPP overexpression and the control group was detected by Co‐IP (F). Western blot was performed to detect the expression of FAK‐related proteins in LHPP overexpression cells and LHPP knockdown cells (G). The data are presented as mean ± SD. ^**^ *p* < 0.01, ^***^ *p* < 0.001, ^****^ *p* < 0.0001.

qRT‐PCR results suggested that PTPRF, NLK, WASF2, IQGAP1, IGF1R, and PTPRJ were upregulated after LHPP knockdown, while downregulated after LHPP overexpression at the transcription level (Figures 6A–C). IGF1R plays a significant role in regulating both cellular adhesion dynamics involving interactions at the cell–cell and cell‐surface levels. However, the repercussions of either the downregulation of IGF1R or its pharmacological inhibition on the intricacies of cellular adhesion processes remain inadequately elucidated. Concurrently, it is noteworthy that adhesion receptors exert a regulatory influence on the signaling pathways of IGF1R.^17^ Western blot showed that IGF1R was significantly upregulated in LHPP knockdown GC cells and downregulated in LHPP overexpressing GC cells at the protein level (Figure 6D). Co‐immunoprecipitation (Co‐IP) observed an endogenous LHPP/IGF1R complex, suggesting that LHPP could interact with IGF1R (Figure 6E). Meanwhile, less IGF1R was bound to LHPP after its overexpression (Figure 6F). Cell adhesion mediated by ITGB1 initiates downstream activation of FAK partially facilitated by the activation of IGF1R. Disruption of ITGB1 ligands or inhibition of FAK signaling results in a reduction of leukemia burden across various organs.^18^ Therefore, we aim to investigate the signaling pathway through which LHPP hinders GC cell adhesion. Western blot was used to detect the expression of ITGB1, FAK, p‐FAK, SRC, YAP, and MYC Proto‐Oncogene (MYC). LHPP overexpression led to a substantial reduction in the expression levels of ITGB1, p‐FAK, SRC, YAP, and MYC. Conversely, LHPP knockdown exhibited contrasting effects by causing an increase in the expression levels of ITGB1, p‐FAK, SRC, YAP, and MYC. In both LHPP overexpression and knockdown GC cells, the expression of FAK remained consistent. Consequently, there was an increase in the proportion of p‐FAK, indicating an enhanced activation of FAK (Figure 6G).

### 2.6 Targeting IGF1R reverses the protumor effects rendered by LHPP inhibition

**FIGURE 7:**
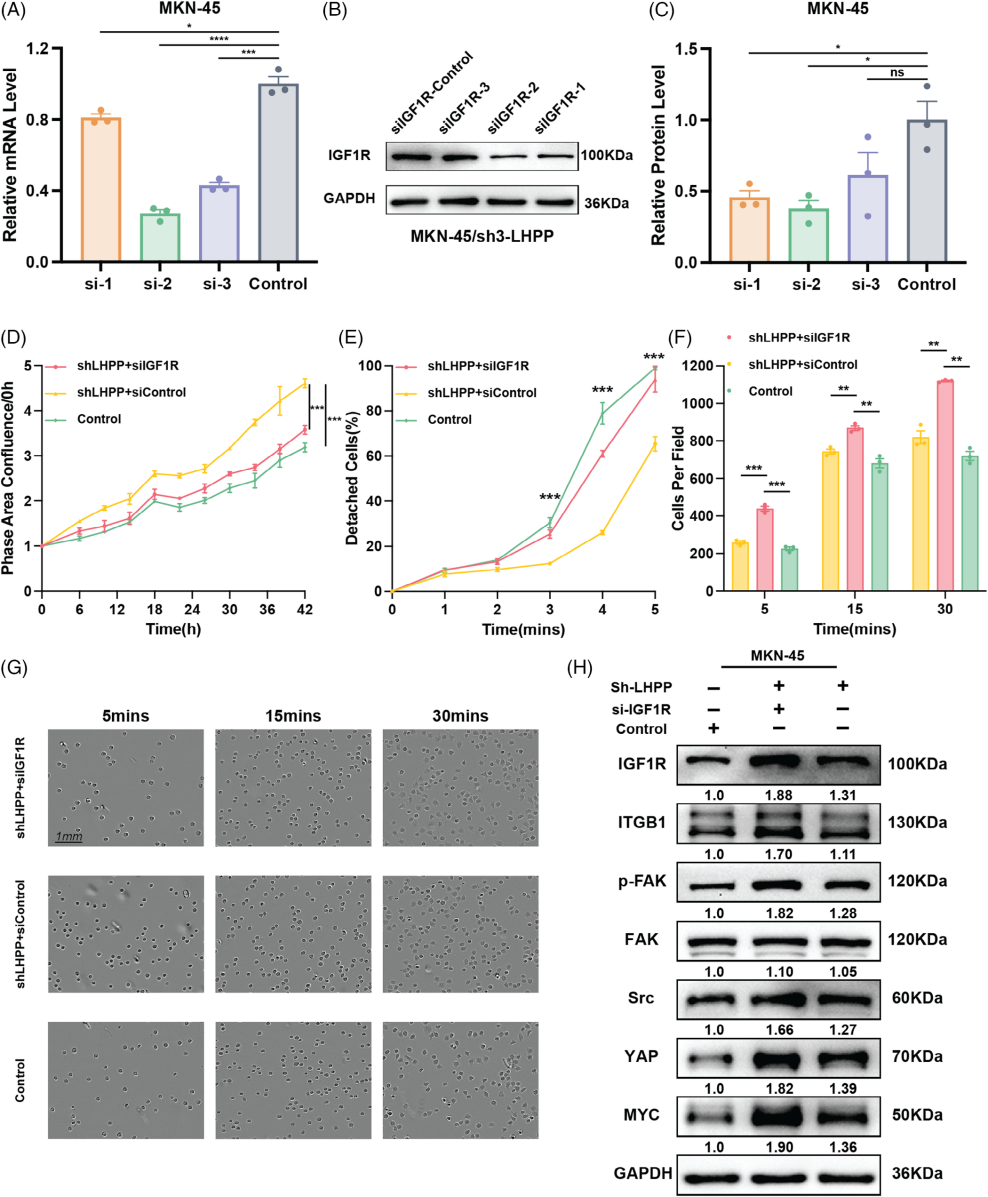
Targeting IGF1R reverses the protumor effects rendered by LHPP inhibition. The efficiency of IGF1R knockdown at RNA level (A) and protein level (B and C) in LHPP knockdown GC cells. Live cell analysis (D), detachment assay (E), and attachment assay (F and G) were performed to detect the cell proliferation and adhesion ability of LHPP knockdown MKN‐45 cells transfected with si‐IGF1R or si‐control. IGF1R regulated proteins expression were detected by western blot in LHPP knockdown cells transfected with si‐IGF1R or si‐control (H). The data are presented as mean ± SD. ^*^ *p* < 0.05, ^**^ *p* < 0.01, ^***^ *p* < 0.001.

Based on the results shown above, the effect of LHPP regulating GC cell adhesion and proliferation might because of IGF1R. To explore the IGF1R function, LHPP‐knockdown GC cells were cotransfected with siIGF1R and corresponding control respectively. Based on the findings from qRT‐PCR and western blot analyses, siIGF1R‐2 demonstrated the most effective knockdown, resulting in IGF1R expression at 40% of that observed in the control group (Figures 7A–C). MKN‐45 cells were cotransfected with sh3‐LHPP+si‐IGF1R, sh3‐LHPP+si‐Control, and sh‐Control+si‐Control. Live cell analysis showed that the downregulation of IGF1R in MKN‐45 cells partially hindered the effect of LHPP knockdown on proliferation (Figure 7D). Meanwhile, attachment and detachment assay indicated that knockdown IGF1R in MKN‐45 cells could reverse the effect of LHPP knockdown on cell adhesion ability (Figures 7E–G). Importantly, the elevation in the levels of ITGB1, p‐FAK, SRC, YAP, and MYC observed in LHPP knockdown GC cells could be partially mitigated by reducing the expression of IGF1R (Figure 7H).

### 2.7 Overexpression of LHPP in GC cells inhibits xenograft tumor growth

**FIGURE 8:**
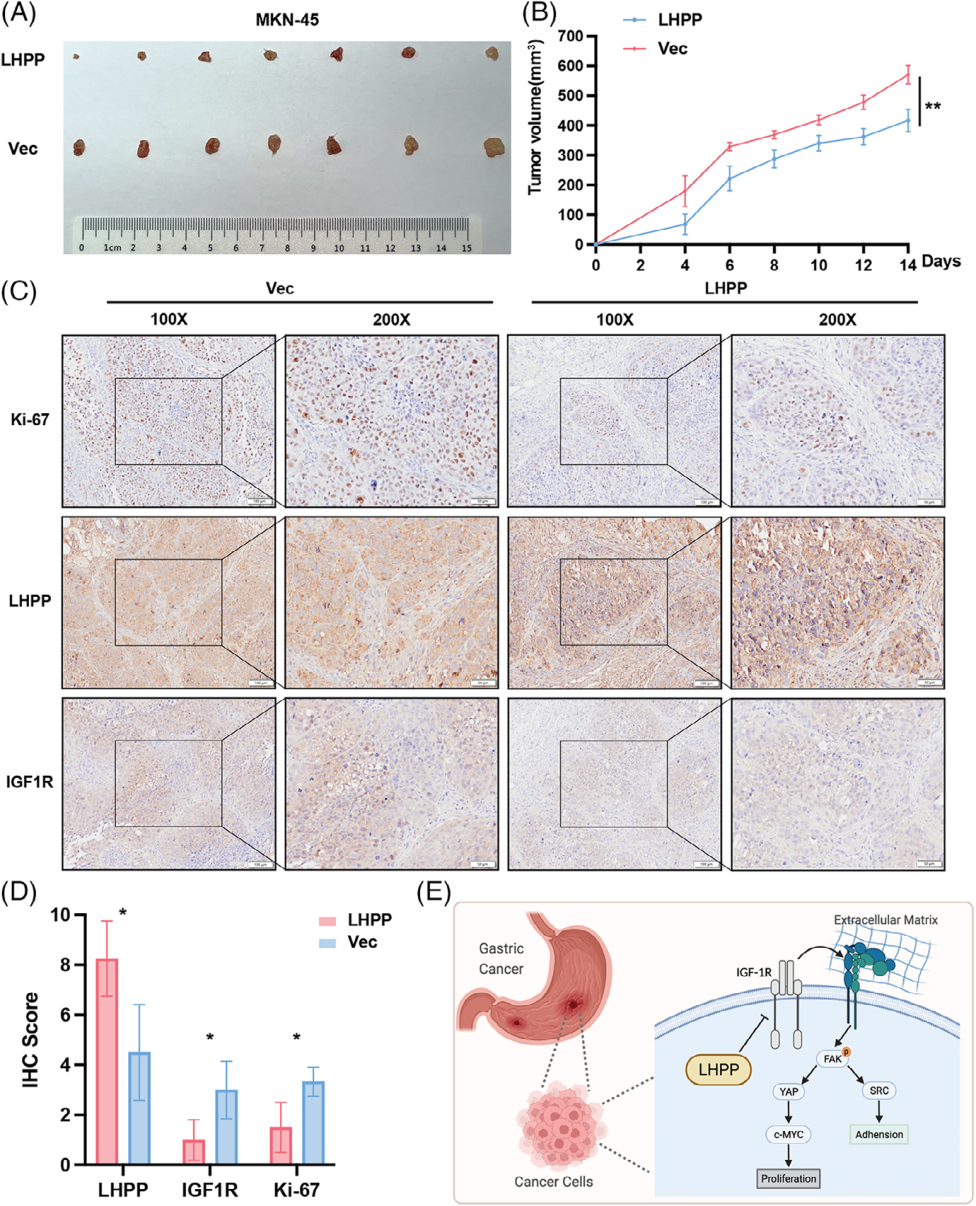
Overexpression of LHPP in GC cells inhibits xenograft tumor growth. Nude mouse models were injected with MKN‐45/LHPP‐OE cells and negative control cells for 2 weeks (A) and tumor volume was measured and analyzed (B). The expression of LHPP, IGF1R, and Ki‐67 in xenograft tumors was detected by IHC (C and D). LHPP downstream regulation pattern diagram (E). The data are presented as mean ± SD. ^*^ *p* < 0.05, ^**^ *p* < 0.01.

To confirm the in vivo impact of LHPP, a xenograft tumor model was established in balb/c nude mice. The xenograft tumors on some mice were too large to have skin ulcers at the top of the xenograft tumors. Therefore, we decided to sacrifice the mice and excise the tumors on day 14 (Figure 8A). Based on the observations and measurements of xenograft tumors every two days, mice injected with MKN‐45/LHPP‐OE cells exhibited significantly constrained tumor growth and volume (Figure 8B). IHC was used to detect the expression of LHPP, IGF1R, and Ki‐67. LHPP were upregulated, while Ki‐67 and IGF1R were downregulated, in the MKN‐45/LHPP‐OE group, suggesting LHPP inhibited GC cell proliferation via downregulation of IGF1R (Figures 8C and D). In general, these results underscored LHPP regulated IGF1R and its downstream pathways to achieve antitumor effects (Figure 8E).

## 3 DISCUSSION

As one of the most common malignant tumors in the digestive tract, GC ranks fifth in global incidence and third in terms of cancer‐related deaths.^1^ Globally, there are approximately 1 million new cases of GC each year, with over half originating from the East Asian region, primarily China.^19^ The uneven distribution of GC incidence may be closely related to genetic mutations, Helicobacter pylori infection, diet, and unhealthy lifestyles.^20^, ^21^ The main treatment modalities for GC are surgery and systemic chemotherapy, and in recent years, targeted therapy and immunotherapy have also made significant progress. While the 5‐year survival rate for early‐stage GC can exceed 70%, the lack of obvious symptoms in the early stages and low prevalence of gastric endoscopy screening often results in patients being diagnosed at an advanced stage.^3^, ^22^, ^23^ Advanced GC tends to metastasize to the liver, peritoneum, or other organs through the lymphatic or circulatory systems, leading to a 5‐year survival rate of around 10% in late‐stage cases. These factors collectively contribute to poor prognosis and unsatisfactory treatment outcomes for GC patients.^2^, ^3^ Therefore, exploring new early diagnostic markers for GC and effective therapeutic targets is of significant importance for improving prognosis and extending overall survival.

LHPP, located on chromosome 10q26.13, was first identified as an important locus for major depressive disorder by sparse whole‐genome sequencing in 2015.^5^ Subsequently, according to genome‐wide association analysis, LHPP was regarded as a susceptibility loci for pharyngeal cancer and childhood acute lymphoblastic leukemia in 2016 and 2017 successively.^24^, ^25^ More importantly, Hindupur et al.^6^ demonstrated that LHPP, a protein histidine phosphatase that was downregulated in tumors, could reduce tumor burden and prevent liver function loss in the hepatocellular carcinoma mouse model. Since then, the role of LHPP as a tumor suppressor gene in various cancers has received great attention. Numerous research elucidated that LHPP promoted apoptosis and inhibited proliferation in various types of cancer by reducing phosphorylated ATK expression and regulating AKT‐related signaling pathways, such as PI3K/AKT, PTEN/AKT, and ATK/AMPK signaling pathways.^7^, ^8^, ^26^, ^27^ Chen et al.^28^ revealed that overexpression of LHPP hindered energy metabolism by degrading PKM2 through ubiquitination in the glioblastoma. Most recently, the TGF‐β/Smad signaling pathway was considered a signaling pathway LHPP participated in to regulate cell migration and invasion in colorectal cancer and intrahepatic cholangiocarcinoma.^29^, ^30^ However, LHPP's significance in GC remains further explored. Therefore, it is of great value to study the role of LHPP in GC. We discovered that the expression of LHPP was lower in gastric tumor tissues than in adjacent normal tissues identified by the TCGA database and 90 GC patients’ samples. Survival analysis showed that GC patients with lower LHPP expression had shorter overall survival. Besides, according to univariate and multivariate COX analyses, LHPP expression might serve as an independent biomarker for predicting GC prognosis. In vitro experiments revealed that GC cell proliferation, migration, and invasion were inhibited after LHPP overexpression, while, enhanced after knocking down LHPP in GC cells, which is consistent with the results in other cancer types, indicating that LHPP functions as a suppressor of cancer initiation and progression in GC.

To further explore the mechanisms by which LHPP impacts GC cells, samples from the Asian Cancer Research Group are categorized into high and low LHPP expression groups. GSEA reveals that differential expression of LHPP significantly affects cell adhesion‐related signaling pathways, biological processes, and molecular functions. RNA sequencing data from LHPP overexpression and control GC cell lines also confirm these results. Cell adhesion is crucial for various cellular functions, and it can be divided into cell–matrix adhesion, which involves interactions between cells and the extracellular matrix, and cell–cell adhesion, which is regulated by epithelial–mesenchymal transition‐related signaling pathways.^31^, ^32^ In the process of tumor metastasis, tumor cells detach from the primary site and implant and grow in other parts of the body via the lymphatic or circulatory systems, leading to dysregulation of both cell–matrix and cell–cell adhesion to some extent.^33^ Many studies have shown that cell adhesion significantly influences tumor cell behavior, including migration and drug resistance.^34^, ^35^ Shao et al.^36^ found that in glioma cells, intersectin 1 (ITSN1) is negatively related to the cell‐to‐matrix adhesion of cancer cells by inhibiting the FAK/Integrin β3 (ITGB3) pathway, thereby inhibiting the progression of tumor. In this study, adhesion and detachment assays unveiled an inverse relationship between LHPP expression and the adhesive capacity of cells. Adhesion ability was improved in LHPP knockdown GC cells, whereas adhesion ability was debased as LHPP expression amplified. Altogether, these results corroborated RNA sequencing results and GSEA analysis and highlight the potential mechanisms underlying LHPP's regulation of GC cell adhesion. Further analysis of RNA sequencing data reveals six differentially expressed genes (DEGs) related to cell adhesion: PTPRF, PTPRJ, NLK, WASF2, IQGAP1, and IGF1R. LHPP is shown to negatively regulate the expression of IGF1R through qPCR and western blot validation.

IGF1R plays a crucial role in cell biology processes such as cell proliferation, metabolism, and tumor development by activating downstream signaling pathways upon binding to insulin‐like growth factor 1 (IGF‐1) or insulin‐like growth factor 2 (IGF‐2).^37^, ^38^ Recently, IGF1R has been reported to promote cancer metastasis and drug resistance.^39^, ^40^ Patients with high IGF1R expression tend to have shorter overall survival and a higher possibility of metastasis in various types of cancer, such as breast cancer, prostate cancer, and lung cancer.^41^, ^42^, ^43^ Pichel and coworkers^43^ found that compared with the normal mouse lung metastasis model, the lung metastasis model of IGF1R‐deficient mice not only inhibited lung tumor proliferation and angiogenesis but also increased cell apoptosis. In hepatocellular carcinoma, overexpressing IGF1R promotes cell proliferation, migration, and antiapoptosis through the PI3K/AKT and extracellular regulated protein kinase pathways, contributing to sorafenib resistance.^44^ In colorectal cancer, IGF1R inhibits the let‐7 microRNA family, promoting malignant progression.^45^ Elevated expression of IGF1R has been found in GC tissues and cell lines, and reducing IGF1R expression can suppress GC cell proliferation and migration.^46^ The negative correlation between LHPP and IGF1R expression is evident in this study. By further experiments involving siRNA knockdown of IGF1R expression, it is observed that the enhanced cell proliferation and adhesion capacity resulting from LHPP knockdown in GC cells are partially counteracted. This suggests that LHPP inhibits GC by suppressing IGF1R expression. We found that IGF1R could promote focal adhesion signaling with ITGB1 and then activate the downstream FAK/SRC signaling pathway to enhance cell adhesion ability.^47^, ^48^, ^49^ ITGB1 encodes the β1 subunit of integrin which is a membrane receptor that regulates cell adhesion and recognition and is involved in many important cellular biological processes such as embryogenesis, hemostasis, tissue repair, immune response, and metastasis of tumor cells. Studies have shown that in prostate cancer cells with decreased expression of IGF1R, the expression of ITGB1 is also significantly decreased. Languino et al.^50^ found that IGF1R stabilizes the expression of integrin by preventing its β subunit from being degraded by the proteasome. FAK is a nonreceptor protein tyrosine kinase that promotes tumor growth and metastasis. It promotes tumor metastasis by regulating the process of tumor cell movement and migration, mainly by regulating tumor cell adhesion and cytoskeleton changes.^51^ In hepatocellular carcinoma, IGF1R activates the downstream SRC signaling pathway to enhance the adhesion ability of hepatocellular carcinoma cells by increasing the phosphorylation level of FAK.^47^ SRC is another nonreceptor protein tyrosine kinase whose expression level is closely related to cell–matrix adhesion and cell proliferation ability.^52^ At the same time, studies have shown that in HCC, ITGB1 can also increase the phosphorylation level to activate FAK, thereby activating its downstream AKT pathway and promoting the metastasis and progression of HCC cells.^53^, ^54^ Therefore, IGF1R can not only activate FAK indirectly through ITGB1 but also directly activate FAK, thereby regulating the adhesion ability of cells. As shown in our results the ITGB1, p‐FAK, and SRC expression were suppressed in LHPP overexpression GC cells, while increased in LHPP knockdown GC cells, which was consistent with former research. Moreover, in LHPP knockdown GC cells, the elevated ITGB1, p‐FAK, and SRC expression were partly reversed by knocking down IGF1R expression. Besides, according to Gutkind and coworkers,^55^ IGF1R could activate YAP through FAK to promote cell growth in triple‐negative breast cancer. YAP, a key molecule in the Hippo signaling pathway, regulated several cell behaviors, including cell adhesion and proliferation.^56^, ^57^ Moreover, the interaction between FAK and YAP had attracted much attention, suggesting FAK promoted cancer development and progression via regulating YAP nuclear translocation through phosphorylation.^58^, ^59^, ^60^, ^61^ YAP acts as a transcription regulator by forming a complex with TEAD. This complex binds to distant enhancers to regulate the expression of their targets.^62^ It is reported that c‐MYC is one of the downstream molecules activated by YAP to promote cell proliferation in liver cancer.^63^, ^64^ Thus, in the present study, YAP and c‐MYC expression were detected via western blot, and we found that the expression of YAP and c‐MYC was suppressed in LHPP overexpression GC cells, while promoted in LHPP knockdown GC cells. This helps explain how LHPP regulates GC cell proliferation through IGF1R.

Certainly, there are some limitations to this study. The main function of LHPP is to remove the phosphate of the catalytic substrate, so the interaction between LHPP and IGF1R is not direct but regulated by intermediate molecules. As shown in our results, LHPP expression could regulate IGF1R, overexpression of LHPP downregulates IGF1R, while knockdown of LHPP up‐regulates IGF1R, at the RNA level. Therefore, it is reasonable to deduce that LHPP might regulate RNA transcription or RNA degradation. Phosphorylation plays a key role in RNA transcription by regulating transcription factors translocation, DNA binding ability, and transactivation.^65^ LHPP could dephosphorylate a transcription factor to keep it from the nucleus or to restrain its ability to bind to IGF1R DNA, as well as transactivation of IGF1R. In terms of RNA degradation, m6A binding proteins and conventional RNA binding proteins are the primary molecules regulating RNA degradation and stability.^66^, ^67^ However, little is known about the relationship between the LHPP and the above two types of proteins. We would like to reveal the regulating mechanism between LHPP and IGF1R in future research. Therefore, additional experiments at the RNA and protein levels, such as RNA pulldown, RNA‐binding protein immunoprecipitation (RIP), Co‐IP, and mass spectrometry, are required to explore the direct target molecules of LHPP, thus perfecting this signaling pathway. Second, the upstream mechanism causing the dysregulated expression of LHPP in GC still deserves further study.

In summary, this study finds that the expression of LHPP is downregulated in GC tissues and cells. LHPP can be used as an independent risk factor for patients with GC, and its expression level is positively correlated with the prognosis of patients. LHPP inhibits the proliferation, migration, invasion, and adhesion of GC cells. The dysregulation of LHPP expression leads to the upregulation of IGF1R, thereby activating the downstream ITGB1–FAK–SRC/YAP–c‐MYC signaling pathway, resulting in the occurrence and development of GC. These findings suggest that LHPP may be a potential diagnostic marker and effective therapeutic target for GC.

## 4 MATERIALS AND METHODS

### 4.1 Bioinformatics analysis

Stomach adenocarcinoma dataset information was acquired from the TCGA database (http://gdac.broadinstitute.org). Clinical patient information was acquired from the GEO dataset (GSE22377, GSE62254), and survival analysis was performed by Kaplan–Meier Plotter (https://kmplot.com/analysis/). Stomach adenocarcinoma samples were sorted ascendingly according to LHPP expression level. Samples from minima to the first quartile and from the third quartile to maxima were defined as LHPP low expression group and LHPP high expression group, respectively. The R software was used to examine the abnormally expressed mRNAs. |log2 (Fold Change)| > 2 and *p* 0.05 were used to screen differentially expnes( DEGs). The substantial enrichment of gene sets and pathways in the obtained dataset was verified using GSEA's functional annotation tool and DAVID Bioinformatics Resources 6.8.^68^

### 4.2 Immunohistochemistry

A tissue microarray (Shanghai Outdo Biotech CO., LTD.) comprising 90 samples of GC and 90 corresponding normal adjacent stomach samples was applied. The tissues were subjected to incubation with a primary anti‐LHPP antibody (dilution 1:500). Subsequently, the resulting staining outcomes were captured using TissueFix. Positive staining of tumor cells was categorized based on staining intensity: 0, 1, 2, 3. The extent of staining was categorized as follows:0, 1, 2, 3. The final expression score (ranging from 0 to 9) was determined by multiplying the proportional staining score by the staining intensity score. Patients in the low‐expression group were assigned final scores of 0, 1, 2, or 3, while those in the high‐expression group received scores of 4, 6, or 9.

### 4.3 RNA sequencing and the following analysis

The RNA Nano 6000 Assay Kit on the Bioanalyzer 2100 system (Agilent Technologies, CA, USA) was employed to assess the total RNA amounts and integrity. For mRNA purification, poly‐T oligo‐attached magnetic beads were utilized. Initial strand cDNA synthesis used a random hexamer primer and M‐MuLV Reverse Transcriptase, followed by RNA degradation using RNase H. After this, the second‐strand cDNA synthesis was carried out using DNA Polymerase I and dNTP. To isolate cDNA fragments in the 370∼420 bp range, library fragments underwent purification using the AMPure XP system (Beckman Coulter, Beverly, USA). Following PCR amplification, the resulting product underwent purification via AMPure XP beads, culminating in the library's formation. Quality assurance procedures were conducted on the library to ensure its integrity. After library construction, its initial quantification occurred through the Qubit 2.0 Fluorometer. Subsequently, the Illumina NovaSeq 6000 was employed to perform library sequencing. Heatmap was visualized by the R package “pheatmap” and gene correlation was analyzed by the R package “corrplot” based on DEGs from RNA sequencing results. DEGs related to adhesion were analyzed by IPA (www.ingenuity.com) for networks and functional analysis.

### 4.4 Cell culture and reagents

GC cell lines, AGS and MKN45 (Guangzhou Jennio Biotech Co., Ltd., Guangzhou, China), were used in vitro experiments. The cell lines were cultured in Roswell Park Memorial Institute (RPMI) 1640 medium (Gibco, Thermo Fisher Scientific, MA, USA) supplemented with 10% fetal bovine serum (FBS; Gibco) in a 5% CO2 incubator at 37°C.

### 4.5 Transfection assay

8 × 10^5^ Cells were seeded and cultured in six‐well plates (Corning Inc., MA, USA) per well at 37°C for 16 h in an incubator. Cells were transfected by LHPP‐overexpression lentivirus (LHPP‐OE) or a negative control lentivirus (LHPP‐NC) and LHPP‐RNAi lentivirus (LHPP‐sh#1/2/3) or a negative control lentivirus (LHPP‐NC) (GeneChem Co., Ltd., Shanghai, China) for 18 h. To create stable and highly pure clones, cells were selected for 48 h in growth media containing puromycin (5 µg/mL; CAS. 58‐58‐2; Beijing Solarbio Science & Technology Co., Ltd., Beijing, China). Cells were transfected by small interfering RNA to knock down IGF1R expression. The efficiency of the transfections was detected after 48−72 h by live‐cell analysis system, western blotting, and RT‐PCR. After transfection, cells were cultured in RPMI‐1640 with 10%FBS and 5 µg/mL puromycin to maintain transfection efficiency.

### 4.6 RNA extraction and quantitative real‐time PCR

FastPure® Cell/Tissue Total RNA Isolation Kit V2 (Vazyme Biotech Co., Ltd., Nanjing, China) was used to isolate total RNA from cells according to protocol. RNA was then reverse transcripted into cDNA using HiScript® III All‐in‐one RT SuperMix Perfect for qPCR (Vazyme Biotech Co., Ltd.). Quantitative analysis was performed via ChamQ Universal SYBR qPCR Master Mix (Vazyme Biotech Co., Ltd.) and the QuantSudio™ 3 real‐time PCR system (Applied Biosystems, Thermo Fisher Scientific) according to the manufacturer's instructions. The expression of GAPDH was employed as an internal reference.

### 4.7 Western blotting

Western blotting was performed as previously described.^69^ The following antibodies and reagents used: primary antibodies against LHPP(#sc‐376648) and Rhoa(#sc‐418) were purchased from Santa Cruz Biotechnology, Inc. (diluted 1:1000; TX, USA); primary antibodies against alpha‐Tubulin(#), GAPDH(#5174), Flag(#14793), YAP(#14074), phosphorylated YAP(S397)(#13619), FAK(#3285), phosphorylated FAK(S397)(#3283), IGF1R(#9750) Src(#2109T), and c‐MYC(#5605) were purchased from Cell Signalling Technology, Inc. (diluted 1:1000; CST, MA, USA); horseradish peroxidase (HRP)‐linked anti‐rabbit IgG (#7074) and HRP‐linked anti‐mouse IgG (#7076) secondary antibodies were purchased from CST (diluted 1:5000).

### 4.8 Co‐immunoprecipitation

The cells were washed twice with PBS. Cell lysates were prepared by adding lysis buffer in a ratio of 20 µL per 1 × 10^5^ cells, with the addition of protease inhibitor (1:100). The mixture was incubated on ice for 10 min and then centrifuged at 14,000 g for 10 min at 4°C. The supernatant was collected. Protein A/G magnetic beads (Protein A/G MCE HY‐K0202) were thoroughly suspended, and 40 µL of beads were resuspended in 400 µL of PBST (1×PBS + 0.5% Tween‐20) twice. Antibodies (LHPP at 1:100, DYKDDDK [CST 14793s] at 1:50, and IgG with the same amount as other antibodies [Beyotime A7016]) were added to 400 µL of PBST, and the magnetic beads were resuspended. The mixture was incubated at 4°C on a rotating mixer for 4 h. After washing the beads four times with 400 µL of PBST, the cell lysate supernatant was added, and the mixture was incubated at 4°C with gentle shaking overnight. Following magnetic separation, the beads were washed four times with 400 µL of PBST, separated, and the supernatant was discarded. To the magnetic beads, 50 µL of 1× SDS‐PAGE loading buffer was added, mixed thoroughly, and heated at 95°C for 5 min, and then the beads were separated. The supernatant was collected for subsequent SDS‐PAGE analysis.

### 4.9 Cell proliferation assay

A total of 5000 cells were seeded in each well of 96‐well plates (Corning Inc.). The IncuCyte S3 platform (Sartorius, Göttingen, Germany) was employed to capture images from various regions within each well at 2‐h intervals, utilizing a 10× objective. The software provided by Essen BioScience (Ann Arbor, MI, USA) was utilized to analyze the cell edges and measure the confluence area of the cells. By comparing the confluence area with the initial images at different time intervals, the relative rate of cell proliferation could be accurately calculated.^70^

### 4.10 Colony formation assay

A seeding density of 300 cells per well was employed in six‐well plates (Corning) for a 14‐day incubation period. After incubation, cells were fixed in PFA (Biosharp, Beijing Labgic Technology Co., Ltd., Beijing, China) for 30 min and stained with 1% crystal violet (Beijing Solarbio Science & Technology Co., Ltd.) for 1 h. Each plate was washed with pure water three times thoroughly before being photographed. Cell number was counted by Image J following protocol.^71^

### 4.11 Wound healing assay

2 × 10^4^ cells were seeded into 96‐well plates to form a monolayer after 8 h of incubation. The monolayer was then lined out using Woundmaker (Sartorius, Göttingen, Germany), with an even gap in the middle. The cells were washed in PBS (Shanghai BasalMedia Technologies Co., Ltd., Shanghai, China) before being cultured in RPMI‐1640 with 5% FBS (Gibco) for 48 h at 37°C in an incubator. Images were taken every 2 h in the middle of the well. The IncuCyte S3 platform (Sartorius) was used to measure the wound distance.^72^

### 4.12 Transwell assay

For the invasion assay, the upper chambers of a transwell plate (24‐well, 8 µm; Corning Inc.) were coated with matrigel (Corning Inc.), but for the migration assay, no matrigel was employed. For migration assay and invasion assay, approximately 10^5^ cells or 2 × 10^5^ cells were plated in the upper chambers and cultured for 24 h, respectively. The lower chamber was filled with 750 µL RPMI‐1640 containing 10% FBS (Gibco), whereas the upper chamber was filled with RPMI‐1640 without FBS. Every chamber was washed with PBS (BasalMedia) three times. After that, chambers were immersed in PFA (Biosharp) for 30 min to fix cells. Chambers were then immersed in 1% crystal violet (Solarbio) for 1 h before three times PBS (BasalMedia) washing procedure. Nonmigrating cells on the upper side of the chamber were gently erased by swab after staining. Under an inverted microscope, cells from three random fields were counted.^73^

### 4.13 Xenograft tumor model

BLAB/c nude male mice (4‐5 weeks old; weight 15−18 g) (Beijing Vital River Laboratory Animal Technology Co., Ltd., Beijing, China) were used as xenograft tumor models in this study. All experimental procedures were conducted following the guidelines and regulations and received approval from the ethics committee of Shandong Provincial Hospital (NSFC: NO. 2021−282). MKN‐45 or LHPP‐OE/MKN‐45 cells were subcutaneously inoculated into the oxter on the right side of the nude mice at a density of 5 × 10^6^ in 0.2 mL of PBS. Tumors were measured every two days by using a caliper and were weighted. The tumor volume was calculated by: [(large diameter) × (small diameter)]^2^/2. Two weeks later, mice were sacrificed, and the tumors were isolated, photographed, and stored at −80◦C for further study.

### 4.14 Adhesion assay

The adhesion assays were performed as previously described.^36^ Matrigel‐coated 24‐well plates (Corning Inc.) were prepared. Subsequently, the cells (at a density of 10^5^ cells/well) were seeded into the Matrigel‐coated 24‐well plates (Corning). After incubation for various time intervals, the cells were washed with cold PBS (BasalMedia). Images of cells adhering to the well were taken by the IncuCyte S3 platform (Sartorius) through an inverted microscope.

### 4.15 Detachment assay

The detachment assays were carried out following the established protocol.^36^ Matrigel‐coated 24‐well plates (Corning) were used for this purpose. Cells (5 × 10^4^ cells/well) were plated in these plates. After 48 h of incubation, the cells were rinsed with PBS (BasalMedia) and then gently trypsinized using 0.25% trypsin (Gibco, Thermo Fisher Scientific, MA) at room temperature. The number of detached cells was quantified at different time points, and the total cell count per well was determined following complete trypsinization.

### 4.16 Statistical analysis

For statistical analysis, GraphPad Prism (GraphPad Software Inc., CA, USA) was employed. A one‐way ANOVA was utilized to compare the means of various experimental groups and applied the least significance different test for post‐hoc analysis. The two‐sided Student's *t*‐test was used to compare the means of two separate groups. Survival curves were constructed using the Kaplan–Meier method and compared with the log‐rank test. Correlation analysis was conducted with Pearson's correlation. In all analyses, probability (*p*) values ≤0.05 were considered statistically significant. ∗, *p* < 0.05; ∗∗, *p* < 0.01; ∗∗∗, *p* < 0.001.

## AUTHOR CONTRIBUTIONS

Wei Chong and Leping Li conceived and designed this study. Liang Shang and Hao Wu analyzed the data. Zihao Zhang performed the research and wrote the draft of the manuscript. Xu Wang and Yuan Liu revised the manuscript. Xingyu Zhu, Chunshui Ye, and Huicheng Ren were responsible for purchasing and providing necessary reagents and consumables. All authors have read and approved the final manuscript.

## CONFLICT OF INTEREST STATEMENT

The authors declare that they have no conflict of interests.

## ETHICS STATEMENT

Animal experiments were approved by the Animal Ethics Committee of Shandong Provincial Hospital (NO.2021‐282).

## Supporting information

Supplementary information

## Data Availability

Sequencing data and clinical annotation of GC samples were retrospectively collected from publicly available datasets of the NCBI‐GEO (https://www.ncbi.nlm.nih.gov/geo/), TCGA (https://cancergenome.nih.gov/). These include those from the GSE62254/ACRG cohort (N = 300), GSE22377(N = 43), and TCGA‐STAD (The Cancer Genome Atlas‐Stomach Adenocarcinoma, N = 375).
