# Supplementary material for: Phospholysine phosphohistidine inorganic pyrophosphate phosphatase suppresses insulin‐like growth factor 1 receptor expression to inhibit cell adhesion and proliferation in gastric cancer
Source: MedComm (2020). 2024 Jan 30;5(2):e472. doi: 10.1002/mco2.472 (PMC10827000; doi:10.1002/mco2.472)
Supplement: Supplementary file 1 — Supplementary information [file MCO2-5-e472-s001.docx]

**Phospholysine Phosphohistidine Inorganic Pyrophosphate Phosphatase Suppresses Insulin-Like Growth Factor 1 Receptor Expression to Inhibit Cell Adhesion and Proliferation in Gastric Cancer**

Author:

Zihao Zhang^1,2^**^#^**, Xu Wang^3,4^**^#^**, Yuan Liu^1^**^#^**, Hao Wu^5,6^, Xingyu Zhu^6^, Chunshui Ye^1^, Huicheng Ren^6^, Wei Chong^1,3,4,6*^, Liang Shang^1,3,4,6*^, Leping Li^1,3,4,6*^

^1^Department of Gastrointestinal Surgery, Shandong Provincial Hospital, Shandong University, Jinan, Shandong, 250021, China.

^2^Department of General Surgery, Zhongshan Hospital, Fudan University, Shanghai, China

^3^Key Laboratory of Engineering of Shandong Province, Shandong Provincial Hospital, Jinan, Shandong, 250021, China

^4^Medical Science and Technology Innovation Center, Shandong First Medical University & Shandong Academy of Medical Sciences, Shandong, 250021, China

^5^Department of General Surgery, Peking Union Medical College, Peking Union Medical College Hospital, Chinese Academy of Medical Sciences, Beijing, China

^6^Department of Gastrointestinal Surgery, Shandong Provincial Hospital Affiliated to Shandong First Medical University, Jinan, Shandong, 250021, China.

**†**: contributing equally

*: corresponding author

Corresponding author:

**Leping Li**

E-mail: [lileping@sdu.edu.cn](mailto:lileping@sdu.edu.cn)

**Liang Shang**

E-mail: [docshang@163.com](mailto:docshang@163.com)

**Wei Chong**

E-mail: [chongwei@sdfmu.edu.cn](mailto:chongwei@sdfmu.edu.cn)

Supplementary Information

Table S1: Univariate analysis of prognostic factors in gastric cancer patients

|  | p.value | HR (95% CI for HR) |
| --- | --- | --- |
| Age | 0.21 | 1.4(0.84-2.2) |
| Gender | 0.85 | 0.95(0.54-1.7) |
| Grade | 0.52 | 1.2(0.74-1.8) |
| Stage | 0.0015 | 2.1(1.3-3.4) |
| LHPP | 0.0064 | 0.52(0.32-0.83) |

Stage (HR:2.1,95%CI:1.3-3.4, p=0.0015) and LHPP expression (HR:0.52, 95%CI:0.32-0.83, p=0.0064) were significantly related to OS according to univariate analysis.
